# Improving membranous urethral length measurements on prostate MRI: a comparison of online training methods

**DOI:** 10.1007/s00330-026-12468-w

**Published:** 2026-03-31

**Authors:** Elisabeth P. Goedegebuure, Thierry N. Boellaard, Margriet C. van Dijk-de Haan, Stijn W. T. P. J. Heijmink, Marinus J. Hagens, Renaud Tissier, Joost J. M. van Griethuysen, Artem Khmelinkskii, Regina G. H. Beets-Tan, Ivo G. Schoots, Doenja M. J. Lambregts

**Affiliations:** 1 Department of Radiology, The Netherlands Cancer Institute, Amsterdam, The Netherlands; 2 GROW Research Institute for Oncology and Reproduction, University of Maastricht, Maastricht, The Netherlands; 3 Department of Urology, The Netherlands Cancer Institute, Amsterdam, The Netherlands; 4 Biostatistics Unit, The Netherlands Cancer Institute, Amsterdam, The Netherlands; 5 Department of Radiology, University Medical Center Utrecht, Utrecht, The Netherlands; 6 The Netherlands Cancer Institute, Amsterdam, The Netherlands; 7 Department of Radiology and Nuclear Medicine, Erasmus University Medical Center, Rotterdam, The Netherlands; 8 University of Deusto, Bilbao, Spain; 9 Department of Radiology and Biomedical Imaging, University of California, San Francisco, USA; 10 Department of Radiology, Memorial Sloan Kettering Cancer Center, New York, NY USA; 11 Department of Radiology, Keio University School of Medicine, Tokyo, Japan; 12 Fondazione Policlinico A. Gemelli IRCCS, Rome, Italy; 13 Spaarne Gasthuis, Haarlem, The Netherlands; 14 Department of Radiology, Addenbrookes Hospital, Cambridge University Hospitals NHS Foundation Trust, Cambridge, CB2 0QQ United Kingdom; 15 IRCCS Ospedale San Raffaele, Vita-Salute San Raffaele University, Milan, Italy; 16 Department of Radiology, University of Cambridge, Cambridge, United Kingdom; 17 Department of Radiology, University College London Hospital NHS Foundation Trust, London, United Kingdom; 18 Centre for Medical Imaging, University College London, London, United Kingdom; 19 Hospital Universitario La Princesa, Madrid, Spain; 20 Department of Diagnostic Imaging and interventional Radiology, S.Maria delle Grazie Hospital, ASL Napoli 2 Nord, Pozzuoli (Na), Italy; 21 Department of Medicine, Surgery and Dentistry, University of Salerno, Baronissi, Italy; 22 Department of Radiology and Nuclear Medicine, Genitourinary Radiology and Mammography, Ghent University Hospital, Ghent, Belgium; 23 Department of Radiology, Rigshospitalet University Hospital, Copenhagen, Denmark; 24 Department of Radiology, Gaziosmanpasa Training and Research Hospital, Istanbul, Turkey; 25 Affidea, Warsaw, Poland; 26 Military Institute of Medicine, National Research Institute, Warsaw, Poland; 27 Radiology Department, San Carlo di Nancy Hospital, Rome, Italy; 28 Department of Diagnostic Imaging, Fondazione IRCCS San Gerardo dei Tintori, Monza, Italy; 29 Medical Termal Center Fontana, Maribor, Slovenia; 30 Hospital Álvaro Cunqueiro, Vigo, Spain; 31 Department of Radiology, IRCCS Azienda Ospedaliero-Universitaria di Bologna, Bologna, Italy; 32 Noordwest Ziekenhuisgroep, Alkmaar, The Netherlands; 33 Department of Radiology, Bedford NHS Trust Hospitals, Bedford, United Kingdom; 34 Institute of Radiology, Department of Medicine, University of Udine, Udine, Italy; 35 University Hospital S.Maria della Misericordia, ASUFC, Udine, Italy; 36 Radiology Department, Unidade Local de Saúde Algarve, Faro, Portugal; 37 Department of Radiology, Franciscus Gasthuis en Vlietland, Rotterdam, The Netherlands; 38 Department of Radiology, Elisabeth-Tweesteden Ziekenhuis, Tilburg, The Netherlands; 39 Fundació Puigvert, Barcelona, Catalunya Spain; 40 Department of Radiology, Prof. Dr. Ilhan Ozdemir State Hospital, Giresun, Turkey; 41 Department of Radiology, Spaarne Gasthuis, Haarlem, The Netherlands; 42 Department of Radiology, Taipei Veterans General Hospital, Taipei, Taiwan; 43 School of Medicine, National Yang Ming Chiao Tung University, Taipei, Taiwan; 44 Radiology Service, Hospital Italiano de Buenos Aires, Tte, Gral, Juan Domingo Perón 4190, Ciudad Autónoma de Buenos Aires, Buenos Aires, 1199 Argentina; 45 Department Thoracic and Abdominal Imaging, University Hospital of Antwerp, Antwerp, Belgium; 46 Department of Uroimaging, Hospital Italiano de Buenos Aires, Buenos Aires, Argentina; 47 Università degli Studi di Napoli “Federico II”, Naples, Italy; 48 Radiology Unit, Department of Surgical and Medical Sciences and Translational Medicine, Sapienza University of Rome – Sant’Andrea University Hospital, Rome, Italy; 49 Department of Radiology, Clinic Favoriten, Vienna, Austria; 50 Clinical Hospital of the University of São Paulo Medical School, São Paulo, Brazil; 51 Department of Radiology, Maastricht University Medical Center, Maastricht, The Netherlands; 52 Department of Radiology, Hospital de Faro, Unidade Local de Saúde do Algarve, Faro, Portugal; 53 Department of Radiology, Catharina Hospital, Eindhoven, The Netherlands; 54 BovenIJ ziekenhuis, Amsterdam, the Netherlands; 55 Department of Radiology, Zuger Kantonsspital, Baar, Switzerland; 56 Department of Diagnostic, Interventional and Pediatric Radiology, Inselspital, University Hospital Bern, University of Bern, Bern, Switzerland; 57 Diagnostic Imaging Service, Hospital Italiano de Buenos Aires, Buenos Aires, Argentina; 58 PixelData SRL, St. Harletului nr.2, Cluj-Napoca, 400423 Romania; 59 Elkerliek Ziekenhuis, Helmond, the Netherlands; 60 Department of Radiology, Catharina Ziekenhuis, Eindhoven, The Netherlands; 61 Department of Advanced Biomedical Sciences, University of Naples “Federico II”, Naples, Italy; 62 Department of Radiology, Laurentius Ziekenhuis, Roermond, the Netherlands; 63 Department of Radiology, Onze Lieve Vrouwe Gasthuis, Amsterdam, The Netherlands; 64 Hospital Clinic de Barcelona, Universitat de Barcelona, Barcelona, Spain; 65 Cambridge University Hospitals NHS Foundation Trust, Cambridge, United Kingdom; 66 ULS São João, Porto, Portugal; 67 Faculdade de Medicina da Universidade do Porto, Porto, Portugal; 68 Department of Radiology, Amsterdam University Medical Center, Amsterdam, The Netherlands; 69 Griffith University, Brisbane, Australia; 70 Royal Brisbane and Women’s Hospital, Queensland, Australia; 71 Body MRI Section, Department of Radiology, Hospital Italiano de Buenos Aires, Buenos Aires, Argentina; 72 Unidade Local de Saúde de Santo António, Porto, Portugal; 73 Department of Radiology, Tartu University Hospital, Tartu, Estonia; 74 Department of Radiology, Ege University Faculty of Medicine, Izmir, Turkey; 75 Department of Diagnostic and Interventional Radiology, Tübingen University Hospital, Karls-Eberhardt University, 72076 Tübingen, Germany; 76 Clinical County Hospital Cluj-Napoca, Cluj-Napoca, Romania; 77 Meander Medisch Centrum, Amerfoort, The Netherlands; 78 Department of Radiology, Ziekenhuis Amstelland, Amstelveen, The Netherlands; 79 Department of Radiology, Reinier de Graaf Ziekenhuis, Delft, the Netherlands; 80 Department of Medical Imaging, Slingeland Ziekenhuis, Doetinchem, The Netherlands; 81 Department of Radiology, The Royal Marsden NHS Foundation Trust, London, United Kingdom; 82 Department of Medical Surgical Sciences and Translational Medicine, Sapienza University of Rome, Sant’Andrea University Hospital-Radiology Unit, AOU Sant’Andrea, Rome, Italy

**Keywords:** Magnetic resonance imaging, Prostatic neoplasms, Urethra, Urinary incontinence

## Abstract

**Objectives:**

This study compared three online training methods to enhance diagnostic performance, reader confidence, and interreader agreement in membranous urethral length (MUL) measurements.

**Materials and methods:**

Ninety-nine study participants were asked to measure the MUL on 20 test cases at the start of the study and rated their confidence level. Participants were then divided semi-randomly into three training groups: Group A (written instructions), Group B (1-month self-study program with case feedback using a dedicated web platform), and Group C (1-day online expert-led case-based training). Groups B and C worked through an identical set of 40 cases. One week after completing training, participants evaluated the same 20 test cases. Diagnostic performance (using expert reference standard), interreader agreement (intraclass correlation coefficient, ICC), and reader confidence levels were compared before and after training. Participants completed a questionnaire evaluating their perceptions of the training.

**Results:**

Eighty-one participants (82%) completed the study. All groups demonstrated significant improvements in diagnostic performance and confidence (*p* < 0.001), with similar outcomes across training groups. Interreader agreement improved from ICC 0.52–0.56 pre-training to 0.74–0.80 post-training. Questionnaire responses indicated most found the training appropriate; however, 17% of Group A felt it was insufficient, while 30–35% of Group C found the expert-led training too time-consuming.

**Conclusion:**

All training methods—ranging from minimal, with written instructions only, to more comprehensive case-based programs—effectively improved diagnostic performance, reader confidence and interreader agreement for MUL measurements on prostate MRI. Independent online training offers practical advantages over more time-demanding hands-on sessions, supporting broader clinical implementation of MUL-based individualized continence prediction.

**Key Points:**

***Question*** *Which method of online training is most effective to train radiologists in measuring the membranous urethral length (MUL) on prostate MRI?*

***Findings*** *81 radiologists showed significant gains in diagnostic performance, confidence and interreader agreement, with similar results after written instructions, case-based self-study, or hands-on expert-led training*.

***Clinical relevance*** *Enhanced accuracy and interreader consistency achieved through online training can facilitate wider clinical adoption of MUL-based continence prediction, strengthening patient counseling and shared decision making in prostate cancer management*.

**Graphical Abstract:**

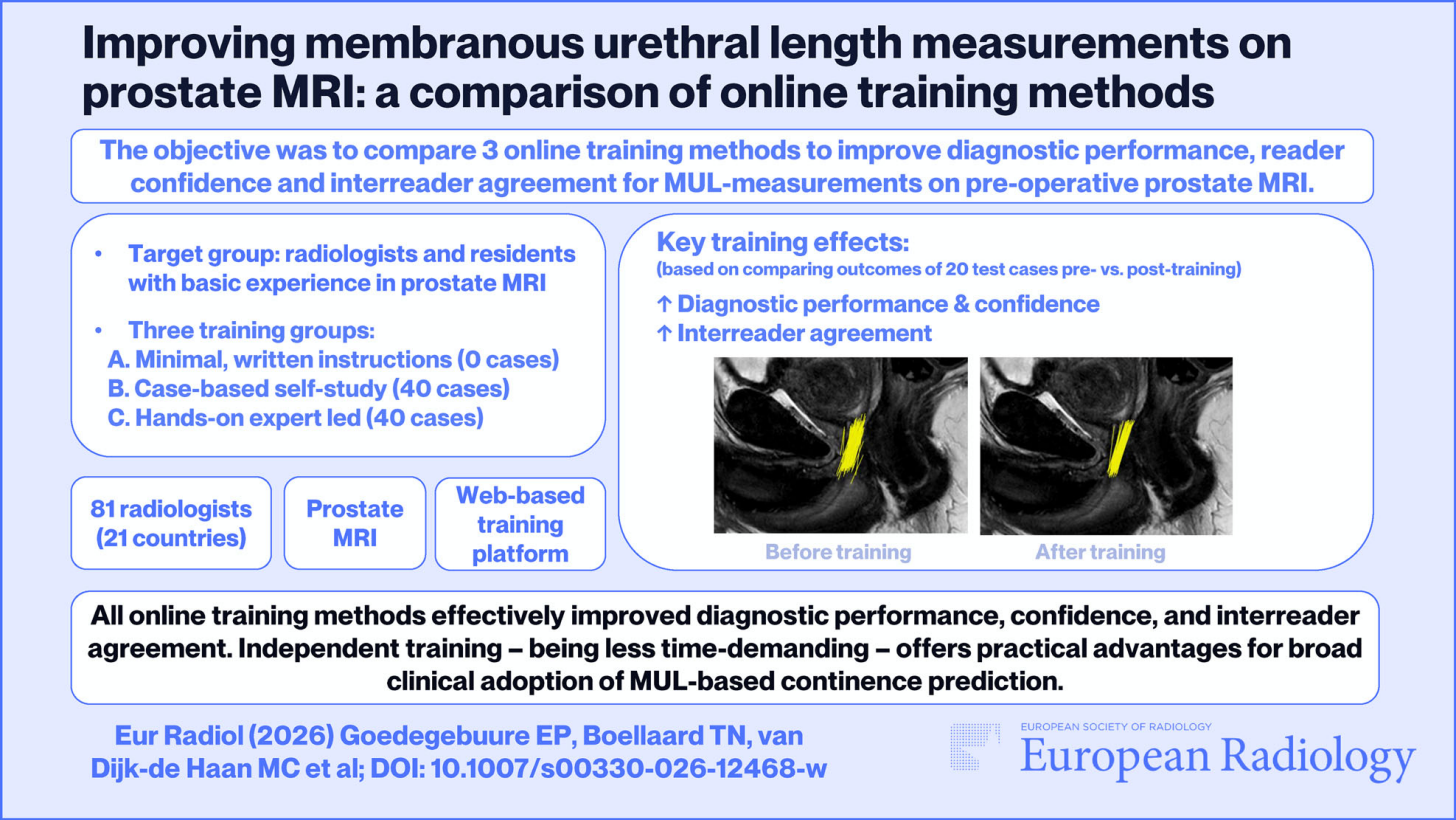

## Introduction

MRI plays an important role in the diagnostic workup of prostate cancer for initial diagnosis, staging and recurrence detection [1]. Depending on clinical guidelines, localized disease may be treated by radiation therapy or radical prostatectomy [2, 3]. Despite its therapeutic efficacy, radical prostatectomy is commonly associated with postoperative complications, including sexual dysfunction and—most notably—urinary incontinence.

A number of pre-operative prognostic factors have been investigated in relation to urinary continence recovery, with anatomical parameters being particularly noteworthy [4]. Among these, the membranous urethral length (MUL)—defined as the linear distance between the inferior margin of the prostatic apex and the superior border of the penile bulb—is by far the most studied MRI parameter, and its predictive power for continence recovery has been demonstrated by several meta-analyses [4–7]. Tillier et al demonstrated that a continence prediction tool incorporating MUL measurements supports more informed, individualized treatment decisions in patients undergoing robot-assisted radical prostatectomy [8]. Despite growing evidence supporting the value of MUL in predicting continence recovery, this measurement has yet to be widely incorporated into routine pre-treatment risk assessment protocols.

For broader clinical adoption, it is crucial to ensure high interobserver reliability and standardization of the measurement technique. Achieving this requires targeted training for radiologists. Previous studies have highlighted the importance of educational interventions in improving radiologic interpretation in prostate cancer as well as other cancers [9–13]. For example, Rosenkrantz et al evaluated the role of independent learning and continual feedback on the learning curve for prostate cancer detection on MRI [12]. They demonstrated a steep learning curve that plateaued after 40 cases, and found that—although availability of feedback did not significantly affect the overall learning curve or accuracy—it did improve reader confidence. Another study by Bregendahl et al in rectal cancer explored the impact of various educational elements on radiologist’s diagnostic staging performance. They showed that it was mainly individual feedback that had a significant impact [13]. These results highlight the value of training with targeted feedback in radiological education. Due to logistical and financial constraints, it is not always feasible to hold in-person training programs, emphasizing the importance of developing effective, accessible online educational strategies.

The objective of the current study was to develop and compare different methods of online training, incorporating varying degrees of targeted feedback, to enhance diagnostic performance and measurement consistency in measuring the MUL on pre-operative prostate MRI.

## Materials and methods

This study concerns a retrospective analysis comparing several training methods for measuring the MUL on pre-operative MRI for prostate cancer. This study was approved by the local institutional review board (IRBd24-268). The need for informed consent was waived.

### Patient selection

Patient cases (20 test, 40 training) were randomly selected from a previous research database described by Veerman et al [14]. The main inclusion criterion was the availability of good-quality T2-weighted sequences in three planes to allow adequate visualization of the membranous urethra. MRIs were performed using a 3-Tesla MR system (Achieva or Ingenia, Philips Medical Systems). From the full protocol, sagittal, oblique-axial and oblique-coronal T2-weighted images (slice thickness 3 mm; voxel size 0.7 × 0.7 mm, reconstructed 0.4 × 0.4 mm) were selected, angled perpendicular and parallel to the peripheral zone-rectal wall border.

### Study participants and training groups

Study participants were recruited between September and October 2024 via open calls sent out to the Dutch Society for Radiology, European Society for Gastrointestinal and Abdominal Radiology, European Society of Urogenital Radiology, and the authors’ professional networks. Radiologists and residents with at least basic experience in reporting prostate MRI were invited to participate. Ninety-nine participants enrolled and were semi-randomly assigned to one of three training groups (ensuring an equal distribution of experience levels between the groups, taking into account prior exposure to the course literature as well as participants’ availability to attend the live training event for Group C). Group A received only written instructions; Group B completed an independent online case-based training; and Group C participated in a hands-on, expert-led training event. Training details are described below.

### Virtual training setup

For this study, a dedicated web-based training platform was used, which combines the Open Health Imaging Foundation (OHIF) DICOM viewing platform with electronic case report forms [10, 15]. The platform was customized for this study by two of the authors (J.v.G., A.K.).

The training setup involved three steps. First, participants completed 20 test cases to establish their baseline (pre-training) performance. For each case, participants measured the MUL and indicated their confidence (unconfident, equivocal, confident). No feedback was provided during this step.

In step two, the groups followed different training protocols:

- Group A (written instructions) independently studied a detailed digital instruction document, based on a previous publication [5], detailing MUL measurement methodology with landmarks, illustrations and image examples. No training cases were provided. One week was given for preparation.
- Group B (self-study) completed 40 training cases over the course of 1 month. For each case, participants measured the MUL and received feedback via an online feedback form containing annotated key images, common pitfalls, and practical tips, as well as reference measurements embedded in the DICOM series (see Supplement 1). The online feedback was generated by a member of the training faculty (M.D.-H.). Reference measurements were based on the average values from two faculty members (T.B., M.D.-H.).
- Group C (expert-led) attended a 6-h interactive online training via Microsoft Teams, led by three expert radiologists (T.B., M.D.-H., S.H.) (8–21 years’ dedicated experience in prostate MRI). After a plenary lecture with Q&A, participants worked in breakout groups (± 10 participants per faculty member). Participants completed the same 40 training cases as Group B, but with real-time guidance. Group C had access to the reference measurements, but did not receive the online feedback forms used by group B. Instead, faculty members reviewed participants’ measurements live to identify errors and challenges. Multiple interactive feedback rounds were incorporated to discuss and correct common mistakes.

Finally, 1 week after completing their training protocols, participants repeated the MUL measurements on the 20 test cases to assess their post-training performance. Additionally, they completed a questionnaire (via Google Forms) to provide feedback on how they perceived their respective training program, and its overall impact on their measurement confidence (rated on a 5-point Likert scale from ‘highly unconfident’ to ‘highly confident’).

### Statistical analysis

Diagnostic performance and interobserver agreement (IOA) were compared pre- and post-training for the 20 test cases. Average measurements from two independent faculty members (T.B., M.D.-H.) served as the reference standard. Diagnostic performance was defined as the absolute difference (mm) between participants’ measurements and this reference. IOA was calculated using the intraclass correlation coefficient (ICC), with ≤ 0 indicating no agreement, 0.01–0.20 slight, 0.21–0.40 fair, 0.41–0.60 moderate, 0.61–0.80 substantial, and 0.81–1.00 high agreement [16]. A mixed-effects linear regression model was used to test the impact of the different trainings on diagnostic performance. To model the relationship between the observations, 2 random effects were used to account for the repeated measurements of each participant and the repeated measurement of each scan pre- and post-training. Covariates included baseline experience (< 10 vs. ≥ 10 years), training group (A, B, C), and an interaction term between course effect and reader experience. A second model did not include experience as a covariate. A third model included resident vs. radiologist as a covariate instead of experience years. Self-reported confidence per case was analyzed pre- and post-training using group as the only covariate. Significance was set at *p* < 0.05. Analyses were performed using R (version 4.3.0): mixed effect regression with *lme4* (version 1.1-32), ICC with *psych* (version 2.3.3).

## Results

### Study participants

**Fig. 1:**
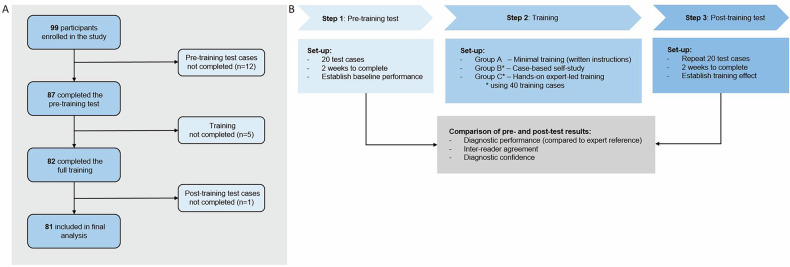
Flowchart showing the inclusion and exclusion of study participants (**A**) and a schematic overview of the study setup (**B**)

The study inclusion flowchart and study setup detailing the three study groups is provided in Fig. 1. Out of the 99 initially enrolled study participants, 81 completed all steps of the study and were included in the final analysis: 29 in Group A, 26 in Group B, 26 in Group C. Included participants originated from 21 different countries, including 14 within and 7 outside Europe. Table 1 summarizes the baseline characteristics of the 81 participants who were included in the study and their distribution across the three training groups. Baseline characteristics, including level of experience, did not significantly differ between the groups (*p*-values ranging between 0.12 and 0.70).

**Table 1:** Baseline characteristics of the 81 participants who completed the study

		Group A	Group B	Group C	Total
Total		29	26	26	81
Sex	Male	19 (66%)	14 (27%)	18 (35%)	51 (63%)
	Female	10 (33%)	12 (40%)	8 (27%)	30 (37%)
Age median (range)		37 (28–58)	39.5 (28–61)	35.5 (30–54)	38 (28–61)
Level of expertise	Abdominal radiologist with specific expertise in prostate MRI	19 (23%)	17 (21%)	13 (16%)	49 (60%)
	Abdominal radiologist	3 (4%)	6 (7%)	6 (7%)	15 (19%)
	General radiologist	3 (4%)	1 (1%)	0 (0%)	4 (5%)
	Residents	4 (5%)	2 (2%)	7 (9%)	13 (16%)
Participation in prostate MDT meetings	Regularly (active participation)	14 (17%)	11 (14%)	8 (10%)	33 (41%)
	Regularly (passive participation)	1 (1%)	1 (1%)	4 (5%)	6 (7%)
	Sometimes	5 (6%)	9 (11%)	8 (10%)	22 (26%)
	Never	9 (11%)	5 (6%)	6 (7%)	20 (25%)
Type of hospital	Comprehensive cancer center	7 (9%)	6 (7%)	8 (10%)	21 (26%)
	General academic center	15 (19%)	7 (9%)	6 (7%)	28 (35%)
	Non-academic center	7 (9%)	12 (15%)	12 (15%)	31 (38%)
	Other	0 (0%)	1 (1%)	0 (0%)	1 (1%)
Years after completion of radiology training	N/A (Residents)	4 (5%)	2 (2%)	7 (9%)	13 (16%)
	< 5 years	13 (16%)	7 (9%)	7 (9%)	27 (33%)
	5–10 years	8 (10%)	7 (9%)	6 (7%)	21 (26%)
	> 10 years	4 (5%)	10 (12%)	6 (7%)	20 (25%)
Estimated no. of prostate MRIs reviewed annually	< 100	5 (6%)	6 (7%)	10 (12%)	21 (26%)
	100–400	17 (21%)	14 (17%)	9 (11%)	40 (49%)
	> 400	7 (9%)	6 (7%)	7 (9%)	20 (25%)
Previous experience with measuring the MUL when reporting prostate MRI	N/A (I do not routinely report prostate MRI)	1 (1%)	2 (2%)	2 (2%)	5 (6%)
	Never	16 (20%)	9 (11%)	7 (9%)	32 (40%)
	Hardly ever	2 (2%)	2 (2%)	4 (5%)	8 (10%)
	Sometimes	3 (4%)	1 (1%)	4 (5%)	8 (10%)
	Most of the time	3 (4%)	2 (2%)	2 (2%)	5 (6%)
	Always	4 (5%)	10 (12%)	7 (9%)	21 (26%)

### Effect of different training methods on diagnostic performance and interobserver agreement

Table 2 shows the average MUL measurements, their deviation from the expert reference standard (= diagnostic performance), and the agreement (ICC) between participants before and after training in each group. There was a statistically significant improvement in diagnostic performance for all groups (*p* < 0.001). Improvement was consistent across all groups, with no significant differences in diagnostic gain between the groups. A non-significant difference was observed between experienced and less-experienced radiologists, with a slightly higher diagnostic gain for less-experienced radiologists (*p* = 0.13). When comparing residents with radiologists, no statistically significant differences in training effects were observed (*p* = 0.77).

**Table 2:** Measurement averages, differences and IOA results across study groups before and after training

	Average MUL measurement (mm ± SD)		Deviation from expert reference (mm ± SD)*		Interobserver agreement (ICC) (95% CI)	
Group	Pre-training	Post-training	Pre-training	Post-training	Pre-training	Post-training
A—Written instructions	15.70 ± 1.96	16.60 ± 1.38	3.32 ± 2.77	2.23 ± 1.83	0.56 (0.42–0.74)	0.80 (0.68–0.89)
B—Case-based self-study	16.03 ± 2.82	17.31 ± 1.61	3.34 ± 2.97	2.13 ± 1.97	0.54 (0.38–0.72)	0.76 (0.64–0.88)
C—Hands-on expert-led training	15.64 ± 2.18	17.04 ± 1.64	3.33 ± 2.99	2.18 ± 2.02	0.52 (0.37–0.70)	0.74 (0.60–0.86)

* The absolute difference between the participants’ measurements and the expert reference was used as a measure of diagnostic performance

**Fig. 2:**
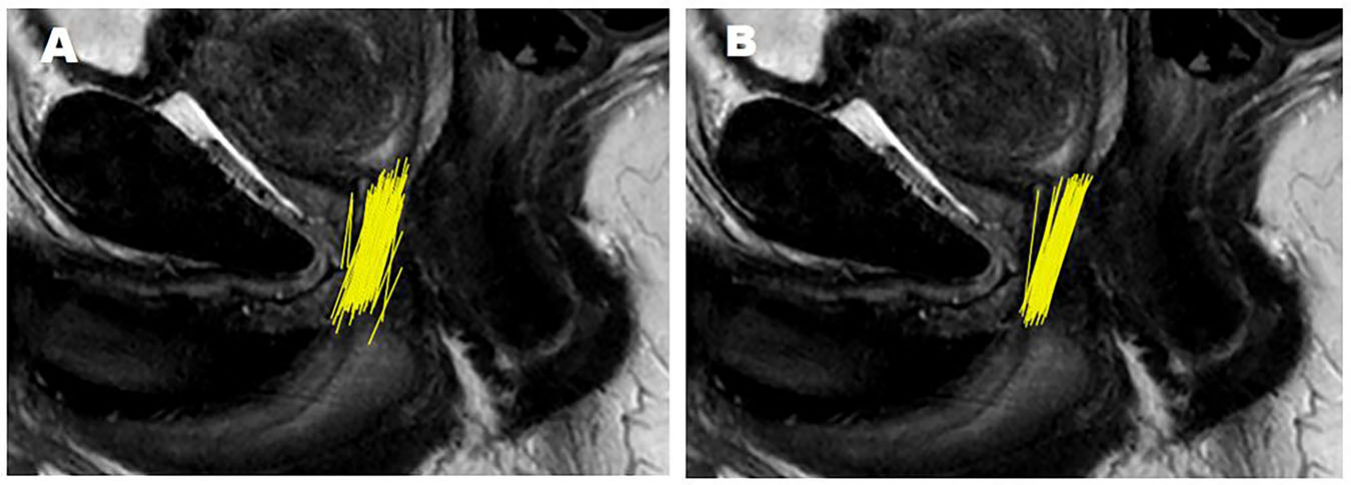
Example of one of the test cases, showing a clear improvement in interreader agreement when comparing the MUL measurements before (**A**) and after training (**B**)

A positive effect was also seen for interreader agreement, with ICCs improving in all groups, from a baseline range of 0.52–0.56 to a post-training range of 0.74–0.80. Agreement between the two experts (whose average measurements served as the reference standard) was excellent, with an ICC of 0.90 (95% CI 0.76–0.96) and a mean (SD) difference of 0.3 (±2.4) mm. 95% limits of interobserver agreement were −4.4 mm and +5.1 mm. A Bland–Altman plot comparing the measurements for the two experts is provided in Supplement 2. A case example showing an improved measurement consistency between readers after training is provided in Fig. 2.

### Reader feedback and diagnostic confidence

**Fig. 3:**
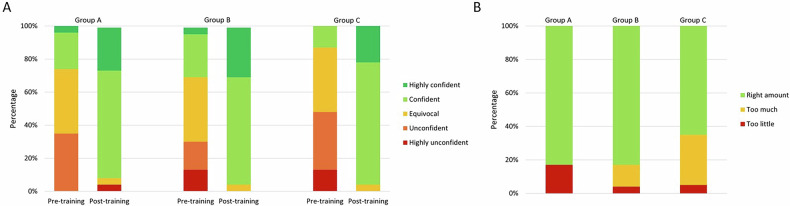
Overview of key questionnaire results illustrating participants’ overall perceived confidence in measuring the MUL pre- and post-training (**A**) and the perceived amount of training (**B**) across training groups

Confidence scores recorded during MUL measurements showed a statistically significant increase across all groups following training (*p* < 0.001). Figure 3 summarizes the results from the post-study questionnaire. Of the 81 participants, 69 completed the questionnaire, accounting for a response rate of 85%. Self-reported confidence in measuring the MUL significantly improved across all groups. When asked about the adequacy of training, most participants across all groups felt they had received the right amount of training. However, 17% of participants in Group A felt they had received too little training, and some indicated they would have preferred some example cases. In contrast, 30% of participants in Group C felt the training was excessive, and 35% noted that the 1-day online training event was too time-consuming.

## Discussion

An international group of 81 radiologists with varying expertise participated in a study comparing three online training methods to improve performance and consistency in measuring the MUL on prostate MRI. Results showed that all training methods—written instructions only, case-based self-study, and hands-on expert-led training—significantly improved diagnostic performance, IOA, and reader confidence, with similar gains across groups. While the amount of training was generally perceived as appropriate, some participants from the written instructions only group desired more case examples, while about one-third of those in the hands-on training group found the training too time-consuming. These perceptions suggest a modest advantage for case-based self-study when perceived confidence is considered, while from a purely performance-based perspective, written instructions alone may be sufficient. No significant differences in performance improvement were observed between more and less-experienced radiologists.

Despite the increased intensity of training in groups B and C compared to group A, no significant differences in training outcomes were found. This may be attributed to the relatively low complexity of the task—measuring a single anatomical parameter. Van Merriënboer et al explored how instructional strategies vary in their effectiveness depending on task complexity [17]. Minimal guidance often suffices for simple tasks, while complex tasks require extensive support, feedback, and structured practice. For simple tasks, too much guidance may boost short-term performance, but hinder independent application later. In contrast, complex tasks place higher cognitive demands on learners, necessitating closer guidance and feedback, with “blocked practice” (repetitive performance of tasks) further enhancing learning. These insights suggest that the straightforward nature of the MUL measurement may have minimized training differences across groups, suggesting a ceiling effect where written instructions may already result in sufficient performance without the need for more elaborate training.

Our findings align with previous studies demonstrating the effectiveness of digital training. For example, Wachsman et al showed improved diagnostic performance in medical students following an interactive, online radiology course, compared to traditional lectures [18]. Similarly, Wang et al found that an integrated approach using simulators, AI-based diagnostic tools, virtual sessions, and small-group learning with structured feedback outperformed conventional training in both theory and practical skills [19]. El Khababi et al developed an online training program for the MRI staging of rectal cancer, including lectures, demonstrations, independent case-based exercises with dedicated feedback, and expert feedback sessions through an online platform [10]. Their results, similar to ours, suggest that online, structured training can effectively replicate the outcomes of traditional face-to-face, hands-on training. Comparable studies in MRI training for pulmonary embolisms and Crohn’s disease have reported similar benefits [20, 21]. A key advantage of online platforms is their capacity to reach a broad, global audience, enabling the large-scale dissemination of educational content and promoting clinical implementation of novel methods. Moreover, the flexibility of online platforms allows participants to engage with the material at their own pace, which may enhance knowledge retention and accommodate diverse learning needs.

The clinical importance of MUL is well established. Longer MUL correlates with faster continence recovery after radical prostatectomy [6, 22], a relationship emphasized in recent clinical guidelines for prostate cancer, which highlight MUL as critical for optimizing postoperative outcomes [2]. Furthermore, it may assist in identifying patients who are most likely to benefit from pelvic floor muscle training, thereby improving early postoperative continence outcomes [23]. Consequently, consistent and accurate measurement of MUL is essential for widespread clinical implementation for personalized postoperative continence prediction. To our knowledge, this study is the first to train a substantial cohort of radiologists and residents to reliably measure the MUL, building upon earlier reports in smaller groups. Lamberg et al showed that specific training improves interobserver agreement among radiologists (ICC 0.62 vs. 0.38 in untrained readers) [24], while Veerman et al similarly reported improved agreement after consensus on measurement technique (ICC 0.84 vs. 0.63) [14].

This study has several limitations. First, no true gold standard exists for MUL measurements; the reference standard was defined as the average MUL measurements of two expert readers, supported by excellent interreader agreement (ICC 0.90). Second, post-training assessments occurred 1 week after training completion, but training durations differed across groups (1 week in Group A, 1 month in Group B, and 1 day in Group C), resulting in variable intervals between pre- and post-testing. Shorter intervals could introduce recall bias, while longer intervals could allow for skill decay. The risk of recall bias was considered minimal, due to the relative interchangeability of prostate MRI cases. Investigating long-term knowledge retention was outside the scope of the current project, but it would be an interesting topic for future research. Group composition also varied slightly: Group A included somewhat more experienced radiologists, whereas Group C had the highest number of residents. These differences were small, not statistically significant, and no significant training differences were observed between residents and radiologists, suggesting that group composition likely did not meaningfully influence the results. Finally, the study used a selected case database with only good-quality images, which may not fully reflect real-world variability in image quality.

In conclusion, this study shows that MUL measurements on MRI can be effectively taught using various online methods. All three approaches—written instructions, independent case-based learning, and hands-on expert-led training—proved effective, with participants showing improved diagnostic performance and increased confidence post-training. Independent training may offer benefits over hands-on training, which is more often perceived as time-intensive. These findings suggest a promising shift toward more accessible and efficient training approaches for radiological skills in clinical practice. Furthermore, the enhanced performance and increased interreader agreement achieved through online training can support the widespread clinical implementation of individualized MUL-based continence prediction, thereby facilitating shared treatment decision making in prostate cancer treatment.

## Supplementary information

ELECTRONIC SUPPLEMENTARY MATERIAL

## Abbreviations

IOA: Interobserver agreement
ICC: Intraclass correlation coefficient
MUL: Membranous urethral length
