# Supplementary material for: Improving membranous urethral length measurements on prostate MRI: a comparison of online training methods
Source: Eur Radiol. 2026 Mar 31;36(8):6419–27. doi: 10.1007/s00330-026-12468-w (PMC13341907; doi:10.1007/s00330-026-12468-w)

Improving membranous urethral length measurements on prostate MRI: a comparison of online training methods

ELECTRONIC SUPPLEMENTARY MATERIAL

Eur Radiol (2026) Goedegebuure EP, Boellaard TN, van Dijk-de Haan MC, et al.

Supplement 1

Example of an electronic feedback file from one of the training cases in Group B (case-based-self-study group). For this group, electronic feedback was integrated into the iScore web-based viewing platform used for this study. Feedback files became accessible immediately upon completion of each case. Additionally, reference measurements were displayed to participants as annotations within the DICOM viewer.

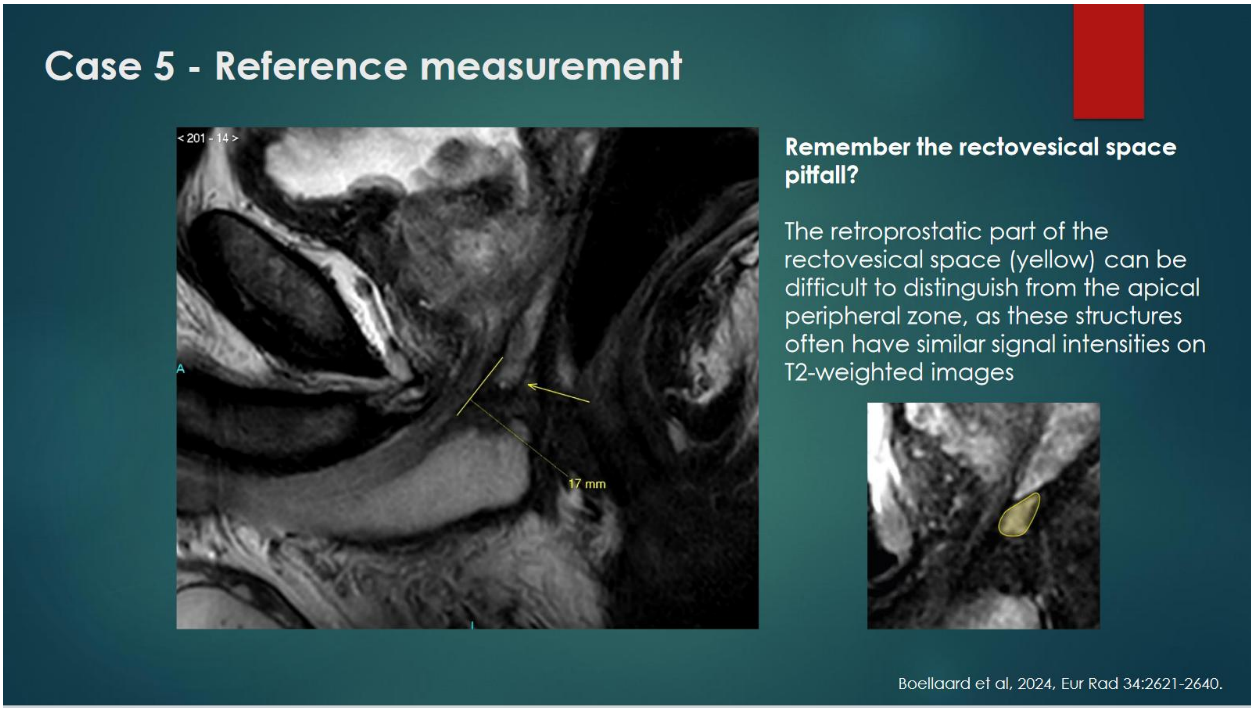

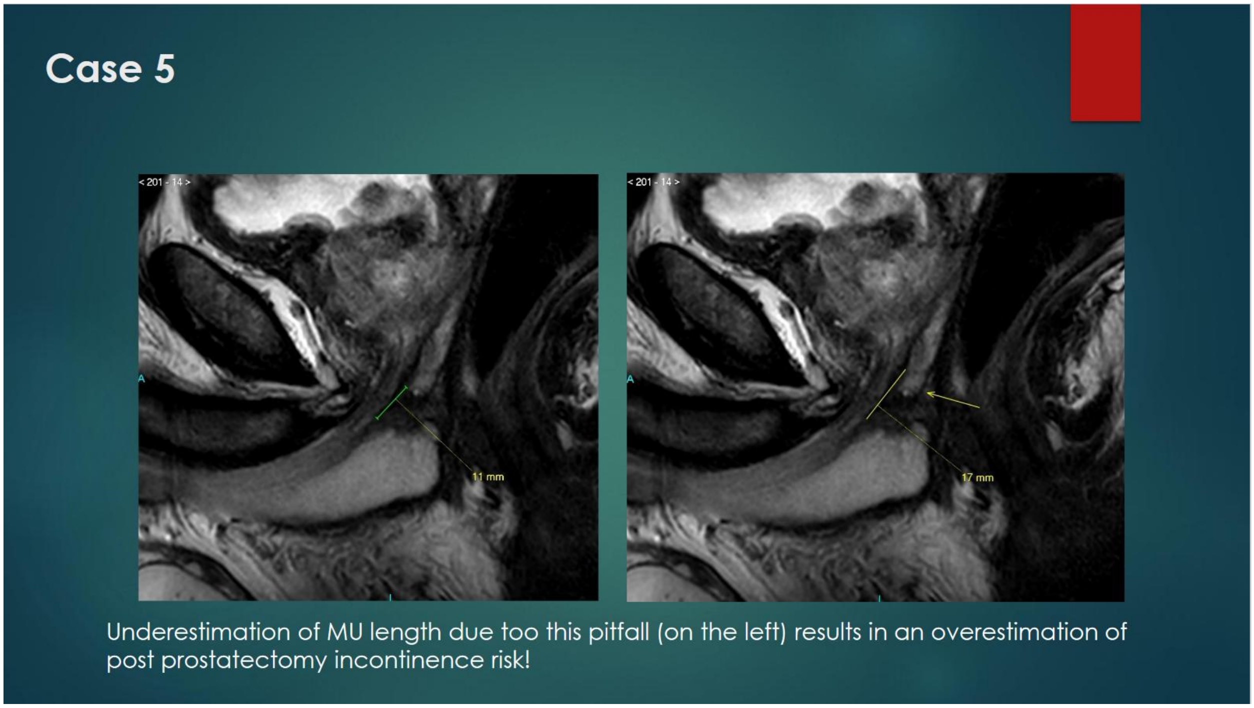

Supplement 2

Bland-Altman plot showing the agreement between the two experts for the 20 test cases. The average of their measurements was used as the standard of reference in this study.

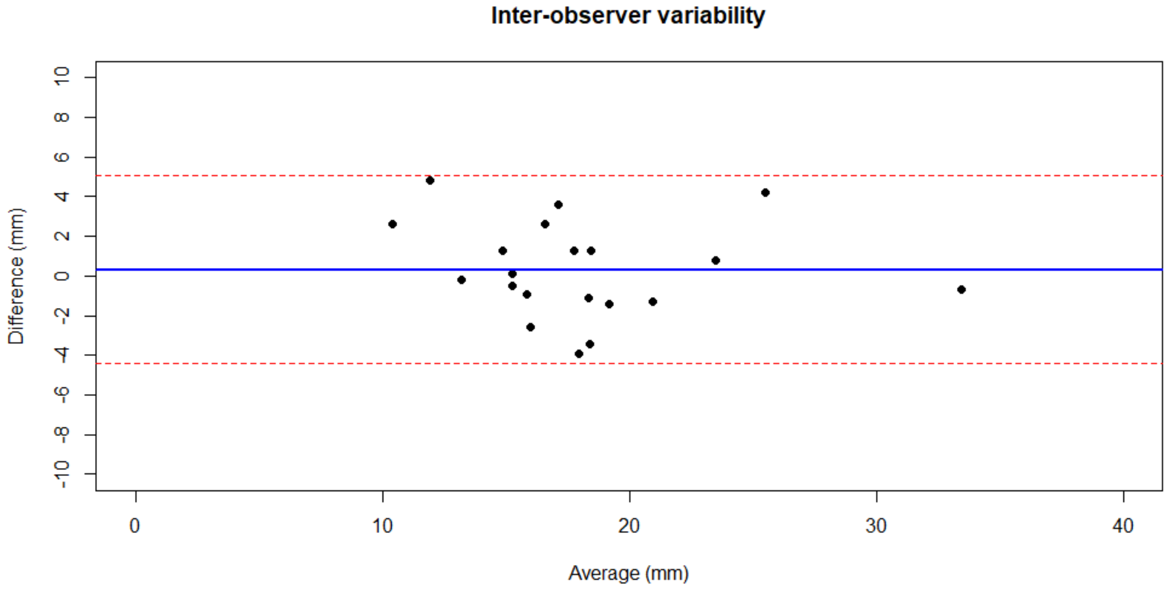

Supplement: Supplementary file 1 — ELECTRONIC SUPPLEMENTARY MATERIAL [file 330_2026_12468_MOESM1_ESM.pdf]
